# Recombinant human epidermal growth factor treatment of radiation-induced severe oral mucositis in patients with head and neck malignancies

**DOI:** 10.1111/j.1365-2354.2008.00971.x

**Published:** 2009-11

**Authors:** JP HONG, S-W LEE, SY SONG, SD AHN, SS SHIN, EK CHOI, JH KIM

**Affiliations:** Departments of Plastic and Reconstructive Surgery, University of Ulsan College of Medicine, Asan Medical CenterSeoul, Korea; Departments of Radiation Oncology, University of Ulsan College of Medicine, Asan Medical CenterSeoul, Korea

**Keywords:** radiation mucositis, rhEGF, head and neck cancer

## Abstract

Mucositis of the oral cavity and pharynx is a major dose-limiting factor in the application of radiotherapy (RT) to patients with head and neck cancer. Therefore, we evaluated the wound healing effect of human recombinant epidermal growth factor (rhEGF) in head and neck cancer and lymphoma patients with irradiation (with or without combined chemotherapy-induced oral mucositis). Patients at Asan Medical Center who had undergone definitive RT of the head and neck region with or without combined chemotherapy and who had developed severe oral mucositis (higher than the Radiation Therapy Oncology Group grade 3) were treated with topical rhEGF twice daily for 7 days. The evaluation of response with regard to oral mucositis was performed 1 week later. Of the 11 treated patients, three had nasopharyngeal carcinoma, three had carcinoma of the oropharynx, two had carcinoma of the oral cavity, one had carcinoma of the hypopharynx and two had lymphoma of the head and neck. Six patients received RT only, and five patients received concurrent chemoradiotherapy. All patients showed improvements in their oral mucositis after topical treatment with rhEGF in that the Radiation Therapy Oncology Group grade was significantly decreased (*P* = 0.0000). This finding suggests that rhEGF is effective and safe for the treatment of radiation-induced mucositis. Further studies are needed to determine the optimal dosage and fractionation schedule.

## INTRODUCTION

Radiation treatment (RT) is the major therapeutic modality in the management of head and neck cancer patients, and the combination of RT and chemotherapy has been shown to have enhanced antitumor effects, which may increase the organ preservation rate (The Department of Veterans Affairs Laryngeal Cancer Study Group 1991; Lefebvre *et al*. 1996; Vokes *et al*. 2000; Fung *et al*. 2005). Although RT combined with chemotherapy may improve the outcome of head and neck cancer treatment, RT combined with chemotherapy, especially with concurrent chemoradiation therapy (CCRT), has been shown to increase the incidence of oral and pharyngeal mucositis, which may restrict this treatment of head and neck cancer (Al-Sarraf *et al*. 1998; Forastiere & Trotti 1999; Fung *et al*. 2005). Radiation-induced injury to the oral and pharyngeal mucosa triggers a cascade of problems, which make it difficult to complete the planned course of RT (Alden *et al*. 1996; Garden 2003). Although interruption of RT results in wound healing, it also allows the tumour cells to recover, with consequent negative impact on the therapeutic effect of RT (Parsons *et al*. 1980; Alden *et al*. 1996). Severe oral or pharyngeal mucositis is very painful, limiting oral intake and resulting in prolonged malnutrition, which decreases the quality of life for these patients and, in rare instances, results in death (Teo *et al*. 1996; Dische *et al*. 1997; Carter *et al*. 1999; Cengiz *et al*. 1999; Trotti 2000).

Rapidly regenerating tissues are easily damaged by radiation or chemotherapy, as dividing cells are sensitive to genotoxic agents. The turnover time of oral mucosal cells is 5 days in mice and humans, so these cells, susceptible to treatment, induce injury (Dorr & Kummermehr 1991; Dorr *et al*. 1994, 1995, 2002; Garden 2003). While CCRT results in improved local tumour control and survival relative to RT alone, it also results in more severe mucosal damage (Al-Sarraf *et al*. 1998; Calais *et al*. 1999). Currently, symptomatic treatment is usually used, as there is no effective radiation-protective method or therapeutic therapy for radiation-induced mucositis. According to the more than 100 studies reviewed by Plevova (Plevova 1999), there is no agent or method that is uniformly effective in preventing or treating the oral mucositis that results from RT.

Epidermal growth factor (EGF), which was first discovered by Cohen in 1962 (Cohen 1962), is a single-chain polypeptide of 53 amino acids (Savage *et al*. 1972; Gregory 1975; Gresik *et al*. 1979). It is present in various normal tissues and body fluids, including the skin, mucosa, tears, cornea, saliva, milk, semen and fluids secreted by the duodenal glands (Heitz *et al*. 1978; Carpenter 1980; Steidler & Reade 1980; Cohen 1983; Olsen *et al*. 1986; Thesleff *et al*. 1988; Waterfield 1989; Weaver *et al*. 1990; Ino *et al*. 1993; Epstein *et al*. 1997). EGF plays an important role in maintaining tissue homeostasis, as it regulates epithelial cell proliferation, growth and migration. In addition, it has an effect on angiogenesis for the nutritional support of tissues. Thus, EGF has a radical effect on wound healing and tissue generation. Recombinant human EGF (rhEGF) has been shown to enhance the mucosal wound healing process (Noguchi *et al*. 1991; Procaccino *et al*. 1994; Girdler *et al*. 1995), which suggests that it may be effective in the treatment of radiation-induced oral mucositis. Therefore, we evaluated the wound healing effects of rhEGF in 11 patients with radiation-induced oral mucositis.

## METHODS AND MATERIALS

Between March 1999 and December 2001, patients treated with RT to the head and neck area, with or without chemotherapy, at the Asan Medical Center were recruited to the study. The inclusion criteria were severe oral mucositis during RT. EGF (Easyef; DaeWoong Pharmaceuticals, Seoul, Korea) was used in these patients. Patients who had previous RT history in the head and neck region or received other mucosal treatment were excluded in this study. The highly purified rhEGF comprises 53 amino acids and is biologically identical to human EGF. The purpose of this pilot study was explained to all the patients, who provided oral informed consent.

Epidermal growth factor was applied topically to the oral cavity twice daily for 7 days. The dosage of rhEGF was 25 µg/day. Changes in the extent and severity of oral mucositis were evaluated at the 7 days later. Mucositis evaluation was carried out with the unaided eye and was restricted to the oral and soft palate. Oral mucositis was scored according to the Radiation Therapy Oncology Group (RTOG) criteria for acute morbidity of mucosal membranes (Table 1). The spss for Windows ver. 12.0 software was used for data analysis. The paired *t*-test with the 95% confidence interval was used to compare the grades of mucositis before and after EGF treatment.

**Table 1:** Oral mucositis assessment scales of the RTOG (Radiation Therapy Oncology Group)

Grade	Acute mucositis
0	None
1	Erythema of the mucosa
2	Patchy reaction <1.5 cm, non-contiguous
3	Confluent reaction >1.5 cm, contiguous
4	Necrosis or deep ulceration, ±bleeding

## RESULTS

Eleven patients, nine men and two women, with a median age of 51 years (range 34–70 years), were recruited to the present study. Of these 11 patients, three had nasopharyngeal carcinoma, three had carcinoma of the oropharynx, two had carcinoma of the oral cavity, one had carcinoma of the hypopharynx and two had lymphomas of the head and neck. Five of the patients were treated with CCRT (Table 2). All patients received the planned radiation dose. None of the patients had to interrupt RT because of acute mucositis.

**Table 2:** Patients’ characteristics

Variables	No of Patients (%)
Gender	
Male	9 (73)
Female	2 (27)
Age	
Period	34–70
Median	51
Diagnosis	
Nasopharyngeal cancer	3 (27)
Oropharyngeal cancer	3 (27)
Hypopharyngeal cancer	1 (9)
Oral cavity cancer	2 (18)
Lymphoma	2 (18)
Radiation dose	
Range	39.6–72.0 Gy
Median	68.0 Gy
Concurrent chemoradiotherapy	
No	6 (55)
Yes	5 (45)

**Figure 1:**
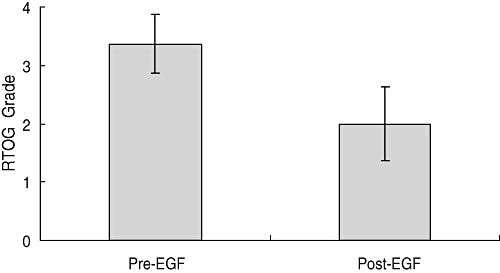
Improved RTOG grades for oral mucositis following topical treatment of the oral mucosa with rhEGF. RTOG, Radiation Therapy Oncology Group; rhEGF, human recombinant epidermal growth factor.

**Figure 2:**
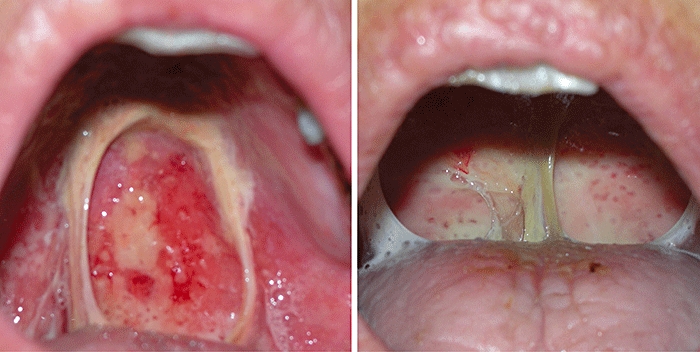
Photograph showing a representative patient with improved oral mucositis. Left panel, RTOG grade 4 mucositis prior to treatment with rhEGF; right panel, improvement to RTOG grade 2 after 1 week of treatment with rhEGF. RTOG, Radiation Therapy Oncology Group; rhEGF, human recombinant epidermal growth factor.

Prior to EGF treatment, four patients had grade 4 and seven patients had grade 3 radiation-induced oral mucositis; their RT doses ranged from 25.2 Gy to 58.2 Gy (median, 32.0 Gy). Following topical treatment with EGF spray for 1 week, all of the patients showed improvements in oral mucositis, with significantly decreased mean RTOG grades (*P* = 0.0000). Of the four patients with grade 4 mucositis, two improved to grade 3 and two improved to grade 2, whereas, of the seven patients with grade 3 mucositis, five improved to grade 2 and two improved to grade 1 (Fig. 1). Figure 2 shows the results for a representative patient who developed RTOG grade 4 mucositis while receiving an RT dose of 48.0 Gy; this patient improved to grade 2 after 1 week of topical treatment with EGF.

## DISCUSSION

Acute mucositis is a serious complication of RT. Mucositis of the oral cavity and pharynx is a major dose-limiting factor for RT in patients with head and neck cancer. The treatment of RT-induced oral mucositis remains a challenge despite several pharmacologic interventions. Interruption of RT because of mucositis prolongs the overall treatment time, which in turn can have a negative impact on local control and may decrease survival rates (Parsons *et al*. 1980; Maciejewski *et al*. 1983; Withers *et al*. 1988; Fowler & Lindstrom 1992; Dische *et al*. 1997). Therefore, the interruption of RT directly correlates with the outcome of RT. In the present study, we show that topical treatment of the oral mucosa with EGF is effective in patients with radiation-induced mucositis. High concentrated EGF spray to the damaged mucosa is a unique compared previous study (Girdler *et al*. 1995). A previous Phase I clinical trial of rhEGF for chemotherapy-induced oral mucositis showed that diluted, low-concentration rhEGF, given as a mouthwash three times daily, delayed the initiation of mucositis and decreased its severity (Girdler *et al*. 1995). In contrast, we sprayed a high concentration of rhEGF directly onto the oral mucosa.

The therapeutic effect of EGF on mucosal damage, including damage to the gastrointestinal tract, has been investigated. EGF has been found to have therapeutic and protective effects against gastric ulcers and intestinal mucositis (Miyazaki *et al*. 1998). As oral mucosa is similar to intestinal mucosa, it seemed reasonable to expect that EGF would exert some effects on oral mucosa. The mucosa is easily damaged by RT or chemotherapy, as the epithelial cells of the mucosa divide very rapidly. In addition, rhEGF has been shown to enhance the healing of chronic diabetic foot ulcers (Hong *et al*. 2006), and we have observed that rhEGF increases the proliferation of human fibroblasts (unpublished observations), which suggest that EGF is effective in various types of wound healing. Based on these biological properties, we tested the topical application of EGF for mucositis induced by anticancer therapy.

Epidermal growth factor stimulates proliferation of various normal and cancer cells. If exogenous provided rhEGF was to stimulate the proliferation of cancer cells, it should not be used in cancer patients because the radiotherapy indication is malignant disease. According to Xia *et al.*, EGF had no stimulatory effect on human gastric cancer cell growth *in vitro* or *in vivo* (Xia *et al*. 2002). In nude mice transplanted with human breast carcinoma, it was shown that rhEGF inhibited the growth of EGF receptor (EGFR)-positive MX-1 cells in a dose-dependent manner, whereas no changes were observed in the growth of cells of EGFR-negative MCF-7, Br-10 or T-61 after treatment with rhEGF. We performed on similar experiment on 25 cancer cell lines and EGF did not stimulate cancer cell proliferation (unpublished data).

Patients with high concentrations of EGF in the saliva have been observed to recover more rapidly from oral mucositis than patients with low concentrations of EGF in the saliva, which suggests that EGF is associated with the healing process in the oral mucosa (Ino *et al*. 1993). The EGF concentration in the saliva is decreased by RT, while the incidence of oral ulcer is increased in patients with low concentrations of EGF (Epstein *et al*. 1997). We believe that topical treatment with EGF accelerates the recovery from mucosal damage caused by RT, with or without chemotherapy, in that EGF is an important protein for mucosal wound healing and cell proliferation is an important factor for the healing of the oral mucosa. EGF is one of the factors involved in the complex process of wound healing.

Several other growth factors, including keratinocyte growth factor (KGF), fibroblast growth factor and transforming growth factor (TGF), are also involved in mucosal wound healing (Wymenga *et al*. 1999; Booth *et al*. 2000; Dorr *et al*. 2002; Spielberger *et al*. 2004). KGF (Palifermin) has been shown to heal mucosal wounds in animal models (Dorr *et al*. 2002) and in human studies, and it has been approved by the US Food and Drug Administration (FDA) as a wound healing agent. Furthermore, the effect of KGF has been demonstrated in a prospective randomized phase III study (Spielberger *et al*. 2004). TGF-B3 has been found to reduce radiation-induced cell damage because of its ability to induce G1 phase arrest both in animals (Booth *et al*. 2000) and humans (Wymenga *et al*. 1999). Although different growth factors have been tested as mucosal wound healing agents, the therapeutic mechanism generally involves epithelial cell proliferation. EGF is a candidate agent for wound healing in patients treated with RT or chemotherapy. KGF stimulates cell growth in some cancer cell lines (Ning *et al*. 1998), whereas there is no report of cell growth stimulation by exogenous EGF (Gill & Lazar 1981; Barnes 1982; Imai *et al*. 1982; Kamata *et al*. 1986; Christen *et al*. 1990). We believe that this finding supports the use of EGF in cancer patients. Granulocyte macrophage colony-stimulating factor (GM-CSF) has been found to stimulate the proliferation of hematologic stem cells and keratinocytes, and keratinocyte proliferation can stimulate the wound healing process (Kaplan *et al*. 1992). GM-CSF has been shown to be effective in the treatment of severe mucositis in patients who have undergone bone marrow transplantation (Nemunaitis *et al*. 1995), although it does not reduce radiation-induced mucositis, according to the one prospective randomized study (Makkonen *et al*. 2000).

In summary, the results of this pilot study show that topical treatment with rhEGF has a therapeutic effect on radiation-induced oral mucositis. Randomized, controlled clinical trials are required to substantiate this effect. In addition, determinations of the optimal dose, fractionation schedule and application method for rhEGF require additional studies with larger numbers of patients.
